# Genomic data and the dividual self

**DOI:** 10.1017/S0016672319000107

**Published:** 2019-12-11

**Authors:** Ian McGonigle

**Affiliations:** Nanyang Assistant Professor, School of Social Sciences, School of Biological Sciences (courtesy appointment), Nanyang Technological University, Singapore

## Abstract

In response to a recent commentary (Tigard, in press) on my previous article, ‘The Collective Nature of Personalized Medicine’ (McGonigle, 2016), herein I discuss collective responsibilities and rights in relation to the ethics of genomic data and personalized medicine. I respond to and elaborate on some of the issues Tigard raises and I draw on the anthropological concept of ‘dividuality’ to emphasize the precisely shared nature of genomic data in order to illuminate the ethical complexity surrounding their protection. Overall, I argue that genomic data, by virtue of their distributed and shared nature, necessitate novel approaches for bioethical assessment.

## 1. Introduction

It is a pleasure to respond to the thoughtful commentary of Tigard (in press) regarding my previous article (McGonigle, 2016) in this journal on the collective nature of personalized medicine. I am grateful for the consideration my article has garnered and for the engaged reading it has received. Tigard, the author of the article ‘Changing the Mindset for Precision Medicine: From Incentivized Biobanking Models to Genomic Data’, is principally concerned with the ethical problems of enrolling participants in databases and precision medicine initiatives. The core issues he raises are that precision medicine is distinct from previous organ/blood donation systems and that prior incentive programmes that worked in the past may not be appropriate for genomic data. The rationale underlying his thesis is that genomic data are qualitatively different from organs, blood or other partible bodily elements. Genomic data may be reused infinitely without being consumed and their immaterial nature makes data a more complex form of property to legally protect and ethically safeguard. Moreover, Tigard correctly suggests that future models for incentivizing participation in databases and biobanks must move ‘toward brand new ways of collectively improving individual treatment and overall health’. Here we certainly agree.

In my previous article (McGonigle, 2016), I made the argument that personalized medicine, despite ostensibly targeting the unique individual, actually depends upon collective participation of large groups in biobanks and genome projects. In that regard alone, personalized medicine is precisely a collective project. That was the major point I wanted to emphasize in my earlier article. In this short rebuttal, I would like to clarify my position on the matter of collective responsibilities and rights, identify the points of agreement with Tigard and elaborate on some of the ethical issues at hand. Principally, I want to draw on the anthropological concept of ‘dividuality’ to further emphasize the precisely shared nature of genomic data. The purpose of this characterization of dividual genomes is to illuminate the ethical complexity surrounding the protection of genomic data.

## 2. The nature of genomic data

We know that genomic data are different in nature from organs. Firstly, as Tigard aptly observes, data are partible and alienable. Secondly, for now at least, ‘vital organs are properly considered scarce and non-renewable resources’ (Tigard, in press). Data and patients’ medical records, however, are abundant even if they are not yet fully available for use in precision medicine databases. Data, in distinction to organs, are highly available and obtainable. Thirdly, in contrast to blood and organs, ‘personal data are very easily duplicated, transmitted and shared with anyone, anywhere.’ Furthermore, when data are consumed, they do not diminish in value. This is an important point. It may even be the case that the more genomic data are used for precision medicine projects, the more valuable the data become for further studies and clinical applications. There is indeed a growing market valuation of data.

There is a further characteristic of genomic data, however, that Tigard did not address: the shared nature that genomic data have with the wider genetic cohort, be they biological kin, tribe, ethnic group or nation. This characteristic makes it difficult and even dangerous to treat individual genomic data as solely and simply individual property and to proceed to protect genomic data under established mechanisms of private property or through systems applied to individual organs. Rather, genomic data need *sui generis* systems of protection and regulation.

## 3. Dividual genomes

Dividuality is an anthropological concept from the study of kinship that describes the intersubjective nature of personhood in contextualized social relations. As opposed to a circumscribed individual self, the dividual self is a distributed entity, relationally constructed, partible, composite and essentially divisible (Strathern, 1988; Wagner, 1991; Gell, 1998; Mosko, 2015). These insights come from the ethnographic study of native communities where the self is understood to exist only within the social networks and situated contexts that lend meaning and identity to the person. The dividual self is also a surprisingly good metaphor for describing the shared nature of genomic data. Like the dividual self in an anthropological sense, genomic data gain their meaning and utility in reference to the wider genomic cohort. Or, more precisely, genetic variants attain their significance in relation to the reference genome.

In thinking of the limits of personal privacy in relation to genomic data, the concept of dividuality is particularly illuminating. Noam Shomron and I first introduced this notion in an earlier article in this journal (McGonigle & Shomron, 2016). There we argued that human genetic personhood (i.e., who you are genetically) and social identity might be better considered as being ‘dividual’ rather than individual, in the sense that genetic data are partially shared with close kin who may also share relevant health and life experiences. Indeed, it is precisely the ‘dividual’ character of genomic data that fosters the establishment of national biobanks and national or ethnic genome projects. A presumed shared set of genetic variants underpins the value in studying a specific ethnic cohort.

The fact that genomic data and the associated personal medical data are precisely ‘dividual’ in nature must perforce impact ethical standards, legislation and governance structures. Legal citizens will have to recognize that when they disclose their perceived personal genomic data publicly, they also share data about their biological kin.

This phenomenon becomes more readily apparent with the growing use and power of genomics in forensics. In 2018, for example, the so-called ‘Golden State Killer’ was arrested in the USA after detectives tracked the suspect down through genetic analysis. The police had previously linked the Golden State Killer to more than 50 rapes and 12 murders from 1976 to 1986, but the investigation had gone cold decades ago. By uploading a DNA sample collected at one of the crime scenes to a recreational genetic ancestry website (Kolata & Murphy, 2018), the suspect was tracked down based on genetic relatedness to participants who had shared their DNA with the genealogy service. In this instance, genetic databases were used for the public good of bringing a notorious murderer to justice. This is just one example of how disclosing personal genomic data may have unintended consequences (positive or negative) for other related persons. But in the fields of healthcare and personalized medicine, there are other bioethical issues to consider when sharing personal genetic data.

Exposing genomic data may impact employment or marriage opportunities. The disclosure of your individual genomic data may entail damages to related individuals who could suffer discrimination as a consequence, particularly if the persons share a high risk of developing an inheritable disease. This potential raises more questions about collective consent, the responsibility or danger in disclosing or restricting data and the limits of personal and family privacy. There arises the issue of balancing individual rights with a collective responsibility to ensure no harm comes to related others. There is thus a potential conflict of interest between protecting individual privacy and the growing importance of genomic databases with the economic valuation of data in the context of personalized medicine projects. Data on specific groups are becoming more sought and valued as we enter the era of ethnic genome projects. Consequently, there are tensions between individual privacy, genomic sovereignty and the need for collective databases for personalized medicine to move forward.

## 4. The genomic group

In the 1990s, there was a call for a worldwide survey of human genetic diversity (Gurwitz *et al.*, 2003, p. 4), initially by the Human Genome Diversity Project. The main assumptions were that the Human Genome Project would not sufficiently capture the human diversity of the world and that there was also a need to better understand the varying degrees of human susceptibility to disease and historical migrations. Since then, national genome projects and national biobanks have proliferated around the world.

After the advent of second-generation genome sequencing, biobanks have also yielded the possibility of cataloguing genomic data on large numbers of people. Fast and high-throughput genomic sequencing platforms raise hopes of revealing the associations of many diseases with single-nucleotide polymorphisms. It is assumed that by identifying the molecular basis for disease a new age of personalized treatment will arrive. One of the principles of personalized medicine is that performing genome-wide association studies with the masses of data generated from thousands of individuals would reveal meaningful disease biomarkers.

These developments have captured the attention of scholars in bioethics and the social study of science and there is a significant literature focusing on biobanks and genomic databases. Much of this scholarship has focused on the ethics of the sampling and storage of human biological material and medical information (Cambon-Thomsen, 2004; Cambon-Thomsen *et al.*, 2007; Haga & Beskow, 2008; Hansson, 2009; McGonigle & Shomron, 2016), the problems and limitations of collective and individual consent (Hansson *et al.*, 2006; Caulfield & Kaye, 2009) and the entailed protection of personal data and the legal definition of the nature of the individual participant (Kaye, 2004; Gurwitz, 2015; McGonigle, 2016). Other work on biobanks has described how transnational collaborations entail challenges for governance where different regulatory and ethical regimes face the challenges of cross-border harmonization (Gottweis & Peterson, 2008; Kaye, 2011; Gottweis & Lauss, 2012; Chen, 2013).

Beyond these pragmatic and normative ethical questions of governance and procedure, biobanks and identity-based genetic research also raise significant and broad-ranging societal concerns that impinge on social identities. Today, social identities (including national, racial or ethnic identities) are progressively attended to in the molecular realm, a phenomenon I have discussed in this journal under the idiom ‘molecularization of identity’ (McGonigle & Benjamin, 2016).

More and more, genetics has entered the lexicon of identity politics. Recently, for example, Lebanese foreign minister Gebran Bassil tweeted about the genetic character of the Lebanese: ‘We have devoted a concept to our Lebanese identity, above any other affiliation, and we have said that it was genetic, since it was the only explanation for our similarity and distinction…’ (Nassar, 2019). Genomics has become a way of imagining collective identity. Indeed, white nationalists in the USA have been wielding genomic evidence to support their racial identity (Panofsky & Donovan, 2019). All of this is to say that genomics has a profound impact on how the ethnic or national group self-identifies, understands itself and defends its boundaries. The significance of this phenomenon in relation to personalized medicine is that genomic data affect not only the individual donor. Genomic data speak for the collective, and accordingly must be ethically recognized as a partially shared resource.

## 5. New ethical directions

Tigard (in press) notes ‘the challenge of amassing the large-scale databases necessary for the success of precision medicine cannot be met by looking to existing models of blood and organ donation wherein contributors are incentivized with distinct personal benefits.’ He is correct. We need to develop novel ways of thinking about the ethics of genomic data that are tailored to their precise nature.

This is all the more important as we enter an era when genomic databasing is intersecting with identity politics. The USA recently launched ‘All of Us’, a research programme that aims to further personalized medicine by generating medical and genomic databases across all US ethnic groups. One of the principles underlying the effort is that genetic variants vary across ethnic groups so that each ethnic group shares certain medical and genetic inheritances. Simply put, you cannot achieve personalized medicine for all ethnic groups without data on all ethnic groups. This is the logic behind the proliferation of ‘ethnic reference genomes’. Such ethnic-based genomic research is flourishing worldwide. GenomeAsia100K, for example, is a Singapore-based human genome project that aims to generate ethnic reference genomes for the major Asian ethnicities. GenomeAsia100K aims to sequence 100,000 Asian genomes in an effort that addresses an ethnic bias towards Western populations in previous genomic research (see McGonigle & Schuster, 2019). It is thus crucial to recognize that genomic data are precisely a shared resource and indeed a shared risk.

## 6. The future of ethnic medicine

As Tigard (in press) observes, personalized medicine may, in fact, become routinized in a way that resembles traditional clinical approaches and older therapeutic models. It is not simply that establishing genomic databases will radically improve patient outcomes. Environmental factors may become more salient in some cases than genetic variants. Nonetheless, personalized medicine will have unique characteristics and will raise novel ethical concerns in each cultural context. In certain cases, we may need to recognize that identifying a genetic variant associated with a disease in a person may impact the patient's biological kin, tribe or ethnic group through stigmatization.

For example, being associated with a lineage that has a particular haplotype with a high disease risk could be detrimental to marriage prospects. This issue is particularly salient in highly endogamous societies where an elevated rate of inheritable Mendelian disorders is present. This is the case in the Arab Gulf countries. The Qatari population, for example, has many inheritable diseases, which have been attributed to a history of tribal endogamy with an estimated consanguinity rate of approximately 54% (Sidra, 2015, p. 47). Consequently, a large-scale genome project, Qatar Genome, has been established to generate genomic data from Qatari citizens.

The goal of Qatar Genome is to reduce the burden of childhood disease associated with such autosomal recessive single-gene disorders. One of the most ambitious aims of the plan is to ‘do whole genome sequencing (WGS) of 10,000 Qataris (3% of the Qatar Genome project)’ (Sidra, 2015, p. 52). This target was the largest international genome project of its time when it launched, comparable to the ongoing sequencing project of the Genomics England project^1^ or Singapore-based GenomeAsia100K in terms of the amount of data generated.

The large-scale genomic sequencing of Qatar Genome has already yielded a high-resolution characterization of the Qatari genome structure (Fakhro *et al.*, 2016). This is the first Qatar ethnic reference genome, which is being used to develop neonatal screening and assessment of genetic disorders prevalent in Qatar. The Qatar reference genome is a map of the rare and common genetic variants in the Qatari population. One of the other achievements of Qatar Genome with Weill Cornell Medicine – Qatar is the development of the first population-specific screening array – the Q-Chip – that can be used to determine risk for recessive disorders known to segregate in the population. The Q-Chip contains the gene variants specific to the Qatari population so that clinical diagnosis of genetic diseases using the Q-Chip will be based on the unique genetic information derived from the Qatari population (Qatar Genome, 2018).

Such ethnic personalized medicine developments as Qatar Genome take us closer to eliminating inheritable genetic diseases, but they also pose ethical dilemmas: who wants to have custodianship of such highly sensitive data? Who is liable if there is a data leak? Is it a crime to expose another person's genomic profile? Or, can you demand that a relative keep secret an inheritable disease for fear of family stigma?

These questions may only be tackled properly if we first recognize the collective – indeed dividual – nature of genomic data. The path forward must entail a reconfiguration of the notion of genomic data as being individual (like organs or blood) to being precisely dividual, as complex and fraught as that may initially seem.

## 7. Conclusion

In conclusion, I concur with Tigard (in press) that genomic data are qualitatively different from other forms of biological substances like blood or internal organs. However, I would add that genomic data may be further distinguished by their ‘dividual’ character, demanding recognition of their shared identity with related others. This phenomenon poses methodological challenges for overcoming the fear of exposure on the side of individuals with certain inheritable disease in their family or wider kin group. It also poses an ethical dilemma as to how to regulate the protection of genomic data. In conceiving of a way forward from these complex ethical dilemmas, I agree with Tigard that new and creative avenues need to be pursued. Crucially, however, I add that we must start thinking of genomic data as a common pool of information and value. Unlike bodily organs, which are clearly defined objects of individual origin, genomic data are far blurrier.

## Footnotes

1 Genomics England was set up in 2015 to deliver the 100,000 Genomes Project, its flagship project to sequence 100,000 whole genomes from NHS patients with rare diseases and their families, as well as patients with common cancers. In late 2018, Secretary of State for Health and Social Care, the Rt Hon. Matt Hancock MP, announced plans to sequence five million genomes over the following five years (see Genomics England, 2018).
